# Molecular mechanisms of mitochondrial uncoupling: focus on 2,4-dinitrophenol

**DOI:** 10.1007/s00249-026-01827-6

**Published:** 2026-03-16

**Authors:** Elena E. Pohl, Olga Jovanović

**Affiliations:** Physiology and Biophysics, Department of Biological Sciences and Pathobiology, University of Veterinary Medicine, Vienna, Austria

**Keywords:** Mitochondria, Uncoupling, Artificial uncoupler, Membrane proteins, Patch clamp, Voltage clamp, SLC25

## Abstract

2,4-Dinitrophenol (DNP) is an artificial mitochondrial uncoupler that was used as an anti-obesity treatment until it was withdrawn due to high toxicity, a narrow therapeutic window, and a lack of an antidote. Despite these concerns, DNP’s efficacy surpasses that of many contemporary anti-obesity drugs, sparking renewed interest in its potential use under controlled conditions. However, the development of antidotes for their toxicity and advancement as therapeutic agents has been impeded by a critical gap in our understanding of the precise molecular mechanisms by which DNP and similar uncouplers operate within mitochondria. This review provides a brief historical overview of DNP use and explores various proposed molecular mechanisms of DNP action. It also summarizes recent results obtained using conductance measurements of artificial bilayer membranes, patch clamp of mitoplasts, in silico analysis, and cryo-electron microscopy. Continued integration of the electrophysiological, structural, and computational approaches is expected to clarify the molecular basis of DNP-mediated uncoupling, enabling the rational development of safer uncouplers and mechanism-based antidotes.

## Introduction in mitochondrial uncoupling

### Strategies for treating obesity

Obesity is one of the most pressing health issues in developed countries worldwide. The most recent report from the World Health Organization (WHO), published in 2022, shows that approximately 2.5 billion adults aged 18 years and older were overweight, including over 890 million adults living with obesity. About 16% of adults worldwide were classified as obese, marking a significant rise from 8.4% in 1990 (source: www.who.int). Overweight and obesity are estimated to account for approximately 4 million related deaths worldwide each year.

The increasing prevalence of obesity is alarming, as it significantly raises the risk of various diseases, including type-2 diabetes, hypertension, cardiovascular diseases, and certain types of cancer. Additionally, obesity is associated with psychological challenges for individuals, underscoring the urgent need for effective therapies. However, the pharmacological treatment of obesity remains a complex challenge.

**Fig. 1:**
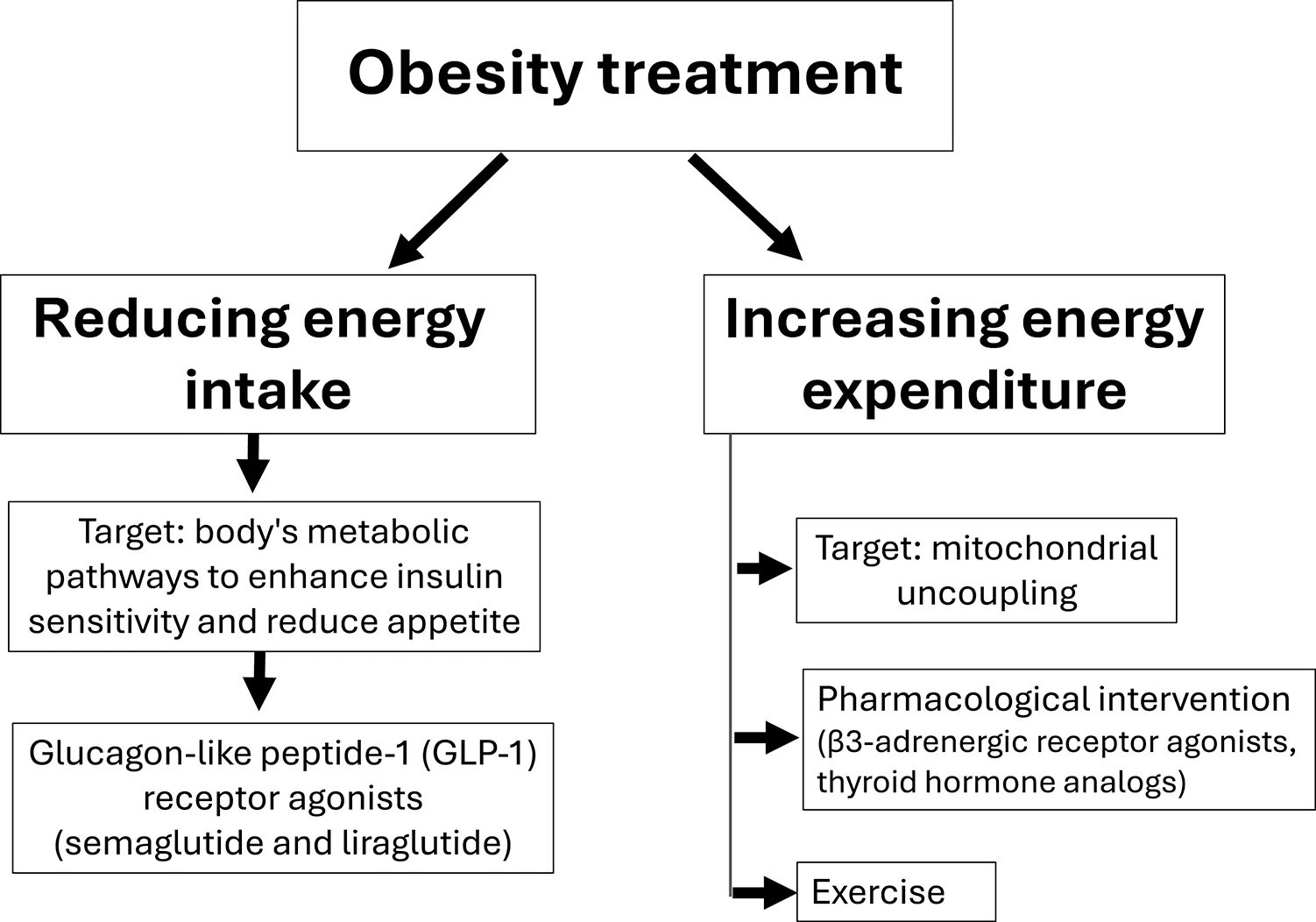
Therapeutic strategies for the treatment of obesity

Therapeutic strategies primarily focus on two approaches: (i) reducing energy intake and (ii) increasing energy expenditure (Fig. 1). The treatment of obesity based on the first mechanism has seen recently significant advancements with the development of anti-diabetic medications that also promote weight loss. These medications primarily target the body’s metabolic pathways to enhance insulin sensitivity and reduce appetite, leading to effective weight management. Glucagon-like peptide-1 (GLP-1) receptor agonists, such as semaglutide and liraglutide, have been approved for both type 2 diabetes and obesity treatment (Scott 2015; Kushner et al. 2020; Watanabe et al. 2024). These drugs mimic the action of the natural hormone GLP-1, which regulates blood sugar levels and appetite and can lead to significant weight loss in individuals with obesity (O’Neil et al. 2018). Sodium-glucose cotransporter-2 (SGLT-2) inhibitors, such as empagliflozin and canagliflozin, primarily used for managing type 2 diabetes, have also been shown to initiate a modest weight loss by preventing glucose reabsorption in the kidneys (Sanjari et al. 2025; Lupsa and Inzucchi 2018; Cefalu et al. 2015). Innovative combination therapies are being developed to enhance weight loss outcomes (Enebo et al. 2021). While these anti-diabetic medications demonstrate efficacy in weight management, they are typically prescribed for individuals with type 2 diabetes and obesity. The utilization of these medications for weight reduction in individuals without diabetes constitutes a domain of ongoing research. Furthermore, clinical trials have demonstrated that both fat and muscle loss can lead to a reduction in overall strength. This phenomenon may be particularly deleterious for elderly patients (Silver et al. 2023; Myerson and Paparodis 2024).

In contrast, anti-obesity strategies based on enhancing mitochondrial uncoupling have shown greater effectiveness, offering a promising avenue for future therapeutic interventions (Goldgof et al. 2014).

### Uncoupling in mitochondria

**Fig. 2:**
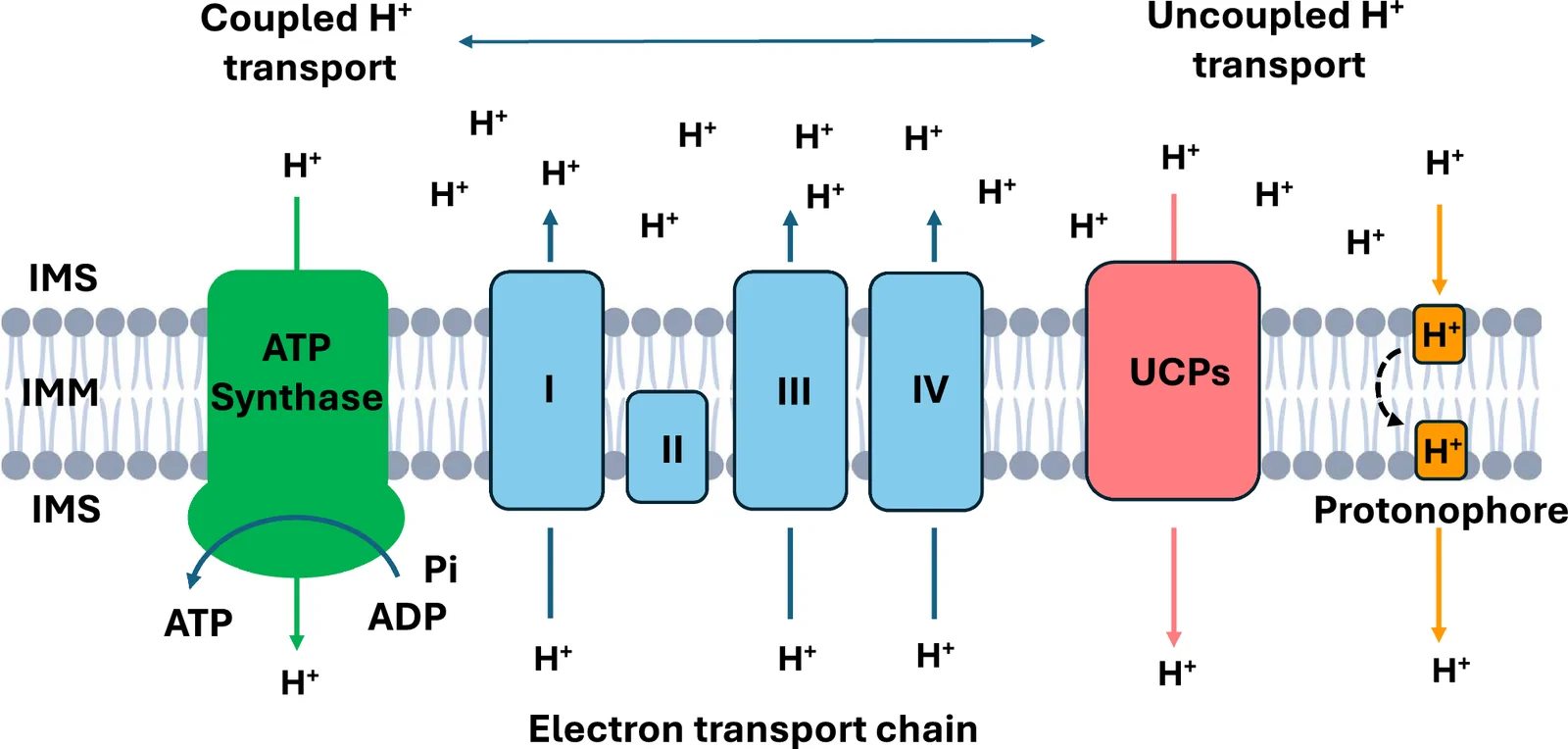
ATP production and uncoupling in mitochondria

Uncoupling is a process that dissipates the proton gradient across the inner mitochondrial membrane (IMM) generated by electron transport chain (ETC) as heat rather than converting it into adenosine-5’-triphosphate (ATP, Fig. 2). Uncoupling is involved in a variety of physiological processes, impacting the balance between energy expenditure and storage, thermogenesis and metabolic efficiency.

Brown adipose tissue (BAT) is essential for adaptive thermogenesis, enabling organisms to enhance heat production in response to cold exposure (Cypess et al. 2025; Nicholls 2023, 2021; Shabalina et al. 2013). In addition to its role in energy dissipation as heat, mild uncoupling can have significant implications for oxidative stress regulation. Mitochondria are a major source of reactive oxygen species (ROS), which are by-products of oxidative phosphorylation (OxPhos). Mild uncoupling has been demonstrated to reduce the generation of ROS, as the proton gradient is less tightly coupled to electron flow in the ETC (Skulachev 1998; Krauss et al. 2005). This phenomenon may provide a protective mechanism against oxidative damage, particularly in tissues exhibiting high metabolic activity.

Dysregulated uncoupling has been implicated in the etiology of several pathologies. In conditions such as obesity and metabolic syndrome, excessive uncoupling can lead to inefficient energy use, contributing to weight gain and insulin resistance.

**Fig. 3:**
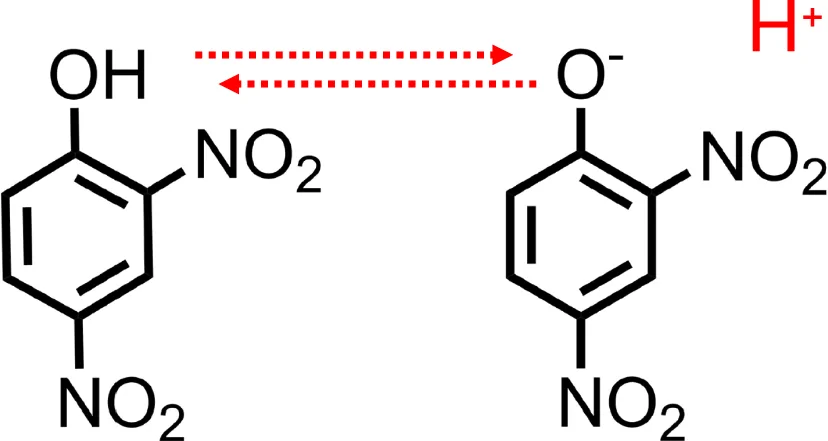
The chemical structure of 2,4-dinitrophenol (DNP) with a protonable hydroxy group

The process of mitochondrial uncoupling is governed by multiple proteins, belonging to the mitochondrial solute carrier 25 (SLC25) family (Pohl et al. 2019, 2025). Uncoupling protein 1 (UCP1) is the most well-known uncoupler, which mediates non-shivering thermogenesis in BAT. The presence of long chain fatty acids (FA), which are weak uncouplers themselves, or other synthetic uncouplers, such as dinitrophenol (Fig. 3), is a *conditio sine qua non* for the uncoupling effect of UCP1. Nevertheless, the precise mechanism through which FAs contribute to UCP1- and ANT1- mediated uncoupling remains a subject of considerable debate (Kreiter et al. 2021; Fedorenko et al. 2012; Roticiani et al. 2025).

### 2.4-Dinitrophenol – an effective but very dangerous synthetic uncoupler

2, 4-Dinitrophenol (DNP; Fig. 3) is an organic compound, (HOC_6_H_3_(NO_2_)_2_, known from the past for its use in the manufacture of explosives, herbicides, wood preservatives, photographic developers, and as an antiseptic or pesticide. In the 1930s, it was marketed as a fat-burning substance, referred to as a “diet pill” (Cutting et al. 1933). The DNP was described to directly increase the metabolic rate by increasing the body’s heat production. However, by 1940, its commercial use was discontinued due to its narrow therapeutic window, which led to severe side effects such as nausea, vomiting, sweating, dizziness, headaches, and in some cases even death. Chronic oral exposure to DNP resulted in cataracts, skin lesions, and damage to the bone marrow, central nervous system, and cardiovascular system (Grundlingh et al. 2011; Siegmueller and Narasimhaiah 2010; Gummesson et al. 2025; Abdelati et al. 2023; Hermetet et al. 2024).

Despite its dangerous side effects, DNP is highly effective in reducing fat while preserving muscle mass. This has led to its resurgence as a “safe weight loss” drug, primarily marketed on the internet and gaining popularity among bodybuilders (Bartlett et al. 2010; Holborow et al. 2016). Not surprisingly, this has resulted in another sharp increase in fatal cases, likely due to uncontrollable hyperthermia (Wise 2014).

The molecular mechanism underlying DNP´s action remains poorly understood, and as a result, no specific antidote exists. The current treatment for DNP poisoning is purely symptomatic and focuses primarily on aggressive cooling measures. Dantrolene is tested to manage hyperthermia in DNP toxicity by reducing muscle excitation–contraction coupling, supporting its potential role in emergency treatment (Kopec et al. 2019).

However, patients often succumb to the effects of the poisoning (Yen and Ewald 2012). The rising incidence of DNP overdose (Gziut and Thomas 2022; Vanthourenhout 2026; Lindeman et al. 2026), coupled with its high mortality risk and the lack of an effective antidote, alongside its potential for promoting weight loss without muscle mass loss and therapeutic uncoupling at low doses, underscores the urgent need to investigate the underlying molecular mechanisms. This could also facilitate the development of safer uncouplers and mechanism-based antidotes.

## Historical overview of the mechanisms behind DNP- mediated uncoupling.

The uncoupling activity of DNP was first described in 1948 by Loomis and Lipmann (Loomis and Lipmann 1948). Subsequent research in the laboratories of Mitchell, Lehninger and Skulachev showed that DNP dissipates the electrochemical gradient generated by respiration in mitochondria and increases proton conductance in black lipid membranes (Bielawski et al. 1966; Skulachev et al. 1968; Mitchell and Moyle 1967a, 1967b). DNP has also been reported to prevent the uptake of inorganic phosphate into mitochondria (Rognstad and Katz 1969).

**Fig. 4:**
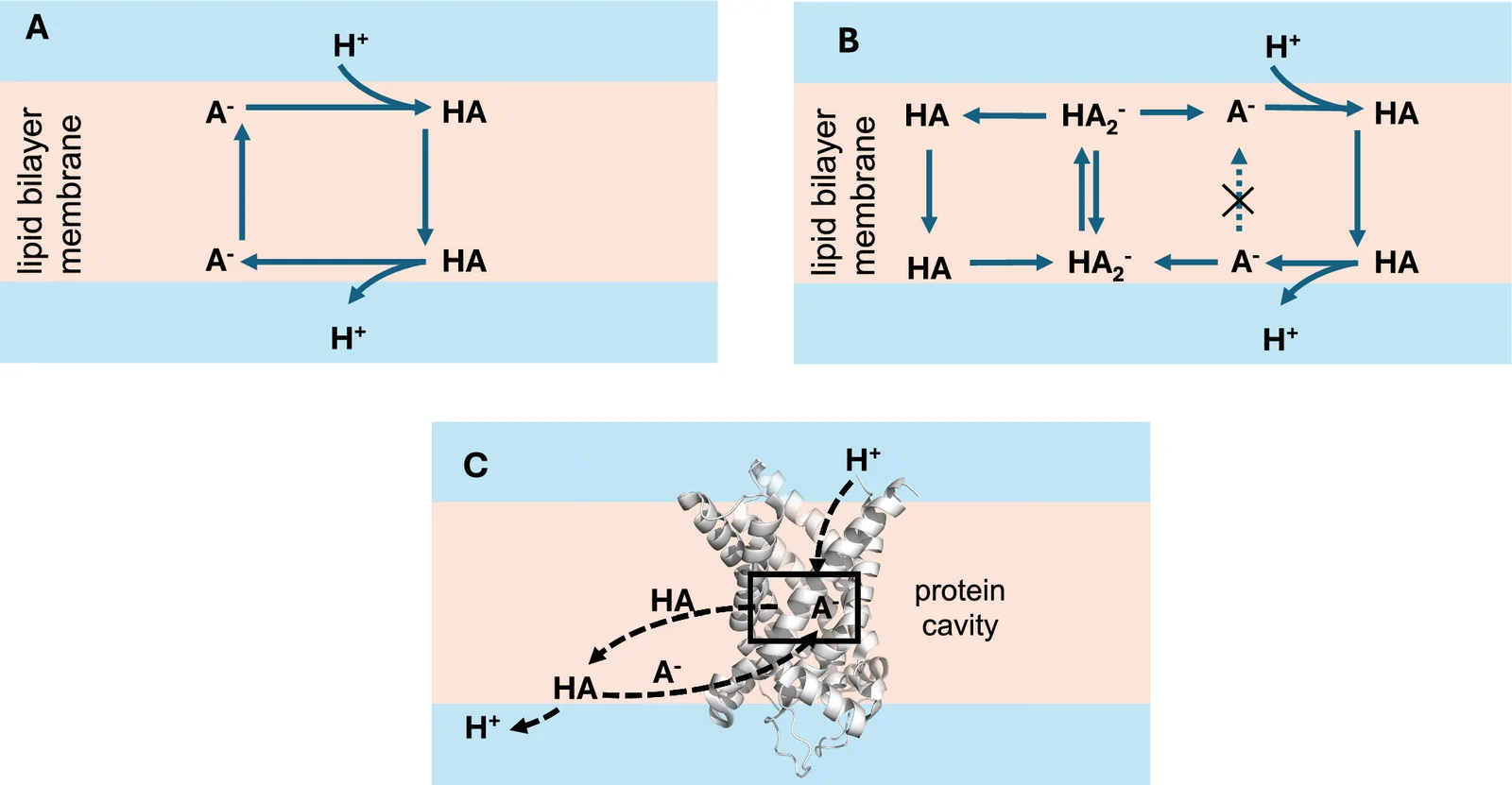
Proposed mechanisms of proton transport across a lipid bilayer membrane, mediated by uncouplers and uncoupling proteins, driven by an applied transmembrane potential or ∆pH. **A**. Protein-independent mechanism by which uncoupler (A^-^) transports hydrogen ion (H ^+^) across lipid bilayer membrane. **B**. Protein-independent carrier model mechanism by which complex (HA_2_^-^) transports uncoupler anion across lipid bilayer membrane. **C**. Protein-dependent mechanism: The mitochondrial protein enhances the transport of hydrogen ions (H^ +^) across the bilayer membrane through an electrostatic interaction with the anionic protonophore (A^-^) in the protein cavity. The dashed arrows illustrate a possible pathway. The illustration shows a cryo-EM model of UCP1 in the DNP-bound state, as obtained by Kang and Chen in 2023.

Generally, substances classified as uncoupling agents do not inhibit specific enzymatic reactions per se (see (Kotova and Antonenko 2022) for a comprehensive review). Instead, they function as lipid-soluble proton donor–acceptor systems that dissolve in the lipid phase of the membrane, where they facilitate the movement of protons across the membrane, thereby disrupting the formation of pH gradients during electron transport (Hopfer et al. 1968; Mitchell 1966) (Fig. 4).

Several groups have attempted to identify the primary target of the molecular mechanism of DNP action. Bielawski, Thompson and Lehninger (1966) were among the first to demonstrate that the conductance of an artificial black lipid membrane (BLM), increased when DNP was used. Hopfer et al. and Liberman/Topaly suggested that the observed increase was predominantly caused by proton transport (Hopfer et al. 1968; Liberman and Topaly 1968). By studying the effect of DNP on (i) the membrane conductance at different pH, and (ii) the impact of pH difference on the transmembrane potential Lea and Croghan (Lea and Croghan 1969) proposed carrier model of lipid-soluble permanent DNP complexes, formed by undissociated DNP and its anion, DNP^−^. Finkelstein proposed the same mechanism for the action of weak acid uncouplers on BLMs, including DNP (Finkelstein 1970). They argued that the negative charge is delocalized across the entire complex in this case, making it much more membrane-soluble than a single anion shown in Fig. 4a. The proposed complex model suggests that BLM conductance increases with the square of the DNP concentration. However, this concentration dependence was not directly observed in the earlier experiments (Bielawski et al. 1966; Liberman and Topaly 1968; Hopfer et al. 1970).

Based on the models of Lea and Croghan (Lea and Croghan 1969) and Finkelstein (Finkelstein 1970), McLaughlin proposed that DNP generates a substantial negative surface potential upon adsorption to a bilayer membrane formed from a neutral lipid (McLaughlin 1972). He provided experimental data supporting his hypothesis of the modified carrier model. However, experiments had to be conducted in the presence of nonactin to demonstrate the effect of DNP. The increase in nonactin-K⁺ conductance produced by the DNP anion was accepted as an adequate indicator of the BLM´s altered surface potential in the presence of DNP anions. When the measured conductance obtained for nonactin was “corrected” for the surface potential produced by DNP anion adsorption, a pronounced quadratic relationship was obtained between conductance and DNP concentration. Unfortunately, this publication did not describe the exact lipid composition of the bilayer membrane. The observed increase in conductance with DNP concentration in the BLM deviates from linear dependence only slightly, and the change in surface potential due to DNP anions is not significantly pronounced for any lipid composition. This could explain why a linear approximation is favored in the literature (Ebert et al. 2018), and why transport of the hydrogen ion (H^+^) by DNP (Fig. 4a) is cited more frequently than the DNP complex model (Fig. 4b). McLaughlin´s work (McLaughlin 1972; McLaughlin and Dilger 1980) lends support to LeBlanc´s broader idea of two classes of weak-acid uncoupler and two mechanisms of charge permeation (Leblanc 1971).

In brief, one class of uncoupler (e.g., CCCP) directly increases membrane conductance in proportion to the protonophore concentration, as illustrated in Fig. 4a. The other class of uncouplers increases conductance in proportion to the square of the uncoupler concentration (Fig. 4b). According to the McLaughlin carrier model, DNP belongs to the second class of uncoupler. These models remain state-of-the art for studying the molecular mechanisms of DNP- and other protonophore-mediated uncoupling in the BLM.

The comparison of the concentration of DNP needed to affect artificial bilayer membranes (approximately 1 mM (Bielawski et al. 1966)) with the much lower concentration required to maximally stimulate respiration in rat liver mitochondria (around 30 µM (Parker 1965)) strongly suggests that proteins may be involved. Hanstein and Hatefi showed that the binding of another potent, water-soluble uncoupler of oxidative phosphorylation, 2-azido-4-nitrophenol (NPA), to mitochondria was saturable and did not appear to involve mitochondrial lipids. Rather, their results indicated the involvement of protein(s) with a molecular weight ranging from 20 to 30 kDa (Hanstein and Hatefi 1974). Experimental evidence of the synergistic effects of dodecadepsipeptide valinomycin and nitrophenolates in lipid membranes may also indirectly support the protein hypothesis (Ginsburg and Stark 1976). However, until recently, no experiments had been performed in systems that allowed for the separation of DNP´s effects on the lipid and protein phases (see Sect. "Recent studies on DNP mechanisms using mitochondria, mitoplasts and lipid bilayers").

## Renewed interest in DNP and its derivatives.

The renewed interest in DNP over the past two decades extends beyond its potential use for anti-obesity treatment. A growing body of recent evidence highlights a variety of beneficial effects associated with DNP application in low doses. Studies have reported that DNP can reverse diabetes, improve hepatic steatosis in rats (Perry et al. 2015), and provide protective effects against neuronal dysfunction and degeneration of neurons in various experimental animal models, including peripheral nerve injury (Madeiro da Costa et al. 2010), a mouse model of multiple sclerosis (O’Neill et al. 2025), traumatic brain injury (Pandya et al. 2007; Hubbard et al. 2023) and ischemic stroke (Chan et al. 2006; Liu et al. 2006). Microdoses of DNP delayed the onset of amyotrophic lateral sclerosis, improved muscle innervation, and reduced inflammation in mice (Zhong et al. 2025). Further results suggest that DNP-induced mitochondrial uncoupling triggers reprogramming of the mTOR and insulin signaling pathways in neurons (Liu et al. 2015). In mice, DNP treatment has been shown to improve learning and memory, consistent with molecular evidence of enhanced synaptic plasticity (Liu et al. 2015). This protection has been attributed to a reduction in mitochondrial free radical production, based on mild mitochondrial uncoupling and/or stabilization of cellular calcium homeostasis.

The growing interest in developing new strategies for DNP applications is further supported by demonstrating controlled release of an oral DNP formulation (Perry et al. 2015; Vlasova et al. 2022). Additionally, the search for suitable DNP derivatives may help to overcome the high, non-adjustable toxicity of DNP (Ost et al. 2017). In combination with the development of an effective antidote, this could make the use of DNP as an anti-obesity drug a realistic and safer therapeutic option. The questions whether the action of DNP is mainly mediated by protein or lipid phase, and the exact molecular mechanism underlying DNP effects remain open (Fig. 4).

## Recent studies on DNP mechanisms using mitochondria, mitoplasts and lipid bilayers

The inner mitochondrial membrane is a hard-to-reach place in the cell. Historically, investigations of DNP´s interaction with mitochondria have been limited to whole cells or isolated mitochondria. These studies primarily measured oxygen consumption, which provides an indirect measure of DNP´s effects since it can be influenced by various mitochondrial processes beyond the proton gradient.

Recently, debates have arisen regarding the effects of DNP and its molecular mechanisms. Studies using artificial bilayer membranes reconstituted with recombinant UCP1, UCP2, UCP3, ANT1, and OGC have shown that these five proteins significantly enhance the protonophoric effect of DNP (see Sect. "1Mechanism of DNP-mediated uncoupling studied using artificial planar lipid bilayer membranes"). Another study confirmed some of these findings using mitoplasts (see Sect. "Patch clamp experiments on the mitoplasts"). The results of these studies were further supported by molecular dynamics simulations (see Sect. "Insights from molecular dynamic simulations"), which revealed that the binding sites for DNP, free fatty acids, and purine nucleotides may overlap.

In contrast, Shabalina et al. examined the effects of DNP on brown fat mitochondria from wildtype and UCP1 knockout (KO) mice and compared them to those of oleate, a known UCP1 activator (Shabalina et al. 2025). As expected, wildtype mitochondria showed higher sensitivity to oleate than UCP1 KO mitochondria. However, oxygen consumption measurements and membrane potential studies revealed no difference in the response to DNP between mitochondria of wild type and KO mice.

While the cause of the discrepancies observed between different systems remains unclear, it is important to explicitly discuss the advantages and limitations of complementary methods in order to correctly interpret and reconcile the results. In Sects. "1Mechanism of DNP-mediated uncoupling studied using artificial planar lipid bilayer membranes" to "Insights from cryo-electron structure of UCP1" of this review, we analyze the results obtained in planar bilayer membranes (Sect. "1Mechanism of DNP-mediated uncoupling studied using artificial planar lipid bilayer membranes"), mitoplasts (Sect. "Patch clamp experiments on the mitoplasts"), in silico systems (MD simulations, Sect. "Insights from molecular dynamic simulations"), and cryo-electron microscopy (Sect. "Insights from cryo-electron structure of UCP1").

### Mechanism of DNP-mediated uncoupling studied using artificial planar lipid bilayer membranes

As discussed in Part 2, BLMs were chosen as the primary model for the investigating the molecular mechanism of DNP action in protein-free membranes. With the emergence of the new protein-mediated hypothesis, using artificial membranes allows to not only reconstitute the protein of interest, but also to compare protein-containing and protein-free membranes in the presence of DNP under the same environmental conditions.

Notably, DNP is not unique in being transported via both protein-mediated and lipid-mediated pathways. A well-established analogy is water transport, which can occur either by passive diffusion through the lipid bilayer or via aquaporins. In such systems, protein involvement generally increases transport rates and enables tight regulation. The involvement of different cellular pathways in DNP transport remains unclear due to the limited number of mechanistic studies of this process. Current evidence suggests that protein-mediated DNP activity arises from binding within protein cavities exhibiting a strong positive electrostatic potential, such as those found in ANT1, which can attract the deprotonated form of DNP. Importantly, this mechanism does not preclude the classical protonophoric action of DNP within the lipid bilayer. Available data (see 4.1–4.4) indicate that both mechanisms coexist in mitochondria. Consistent with this, DNP-induced conductance is substantial in protein-free artificial membranes but increases markedly in the presence of membrane proteins. Given the exceptionally high protein content of the inner mitochondrial membrane (approximately 75% by weight), elucidating the interactions between DNP and membrane proteins is essential for a comprehensive understanding of its uncoupling activity.

Further advantage of the artificial membranes is the possibility to study the properties of a single type of membrane protein within a lipid membrane of well-defined composition. It allows different recombinant mitochondrial proteins to be tested and compared under consistent physicochemical conditions such as pH, temperature, osmolarity, lipid-protein ratio. Consequently, this method is optimal for elucidating the molecular mechanisms underlying the interaction between membrane proteins and their substrates. Site-directed mutagenesis allows for the generation and testing of specific protein mutants under the same conditions as the wild-type protein. This enables the direct evaluation of how individual amino acids impact protein-substrate interactions. However, a major limitation of this method is that the lipid bilayers may not accurately replicate the asymmetry found in natural membranes because the lipids are symmetrically represented in both membrane leaflets.

Using unilamellar liposomes, the significant influence of membrane lipids on DNP adsorption within the lipid bilayer and its effect on DNP-mediated total membrane conductance was demonstrated (Jovanovic et al. 2019). Žuna and colleagues used artificial lipid membranes to test the hypothesis that ANT1, UCP1-3, and OGC facilitate the transport of DNP anions across the lipid bilayer (Zuna et al. 2021; Zuna et al. 2024), in a manner analogous to free fatty acid transport (Kreiter et al. 2021; Kreiter et al. 2023; Andreyev et al. 1989; Bertholet et al. 2019) (Fig. 4C). This hypothesis is based on the observation that DNP- induced mitochondrial uncoupling is sensitive to carboxyatractyloside (CATR), a specific inhibitor of ANT.

Previously, it was known that only long-chain free FAs could directly activate ANT1, OGC, UCP1, UCP2 and UCP3 (Kreiter et al. 2021; Zuna et al. 2024; Zackova et al. 2003; Beck et al. 2006; Beck et al. 2007; Macher et al. 2018). However, DNP was found to be a much more efficient activator of these proteins than FAs. Only 50 µM of DNP produced an effect similar to that of 331 µM of arachidonic acid in artificial planar lipid bilayer membranes (Zuna et al. 2021). Using artificial bilayer membranes, it is possible to directly verify that DNP-induced mitochondrial uncoupling is sensitive to CATR. In a lipid bilayer membrane reconstituted with ANT1, CATR inhibited proton transport by up to ~ 98% at a concentration of 100 µM when added before DNP, indicating specificity of the interaction with ANT1. Since ATP has been shown to inhibit FA-activated ANT1 (Kreiter et al. 2023), the effect of DNP on ANT1 activation was examined. It was found that, at a concentration of 4 mM, ATP inhibited the effect of DNP by ~ 96% when added before DNP. The same was observed for UCP1, UCP2, and UCP3. These results suggest the same putative binding site for DNP, CATR, ATP, and FA in ANT1. Based on previous studies that showed that arginine 79 (R79) is critical for the binding of CATR and ATP to ANT1 (Nelson et al. 1993; Heidkamper et al. 1996; Pebay-Peyroula et al. 2003), the authors tested its role in the interaction of DNP with ANT1. Site-directed mutagenesis was used to generate the ANT1-R79S mutant, in which the positively charged arginine was replaced by a polar serine. Recombinant ANT1-R79S was found to be unable to facilitate proton transport mediated by DNP. These experiments proved and confirmed the hypothesis that several mitochondrial proteins potentiate DNP-mediated proton transport across the membrane. R79 was identified as the putative binding site for DNP in ANT1. However, the involvement of other amino acids is conceivable and deserves further investigation.

The propensity of the DNP anion to interact electrostatically with positively charged amino acid residues—particularly arginine and lysine side chains—suggests that multiple inner mitochondrial membrane proteins may enhance the activity of DNP and related protonophores. Such protein-mediated amplification of uncoupling could help explain the difficulty in halting the uncontrolled heat production observed in cases of DNP overdose, as the compound may engage numerous protein interaction sites rather than a single, targetable pathway.

This is particularly true for proteins such as UCP2, UCP3, and OGC, which are less abundant in mitochondria than ANT1 and therefore cannot be directly measured using the patch clamp method as described in the Sect. "Patch clamp experiments on the mitoplasts".

### Patch clamp experiments on the mitoplasts

Bertholet and colleagues applied patch-clamp techniques to isolated mitoplasts (Bertholet et al. 2022) and showed that DNP induces a proton leak in the inner mitochondrial membrane (IMM) by activating the mitochondrial proteins ANT and UCP1. The main advantages of this method are that it allows for the study of mitochondrial protein functions in their native membrane environment and enables the adjustment the buffer conditions. However, several critical issues remain. The seemingly optimal physiological conditions are questioned because: (i) osmotic pressure is applied to produce the mitoplasts and (ii) the potential present at the inner mitochondrial membrane is destroyed. Furthermore, it is difficult to prove that the correct protein is being measured. Selecting the correct control is challenging. So, Bertholet et al. used mitochondria from skeletal muscles (SkM) to show the lacking protonophoric function of UCP2 (Bertholet et al. 2019), although it was previously demonstrated that no UCP2 is present in SkM (Rupprecht et al. 2014; Rupprecht et al. 2012).

The effect of DNP on UCP1 was studied in mitoplasts isolated from BAT, where UCP1 is the most abundant protein under cold acclimating conditions. It was observed that high concentrations of DNP (500 μM), in combination with an FA scavenger (10 mM β-cyclodextrin), induced a significant proton current in the IMM. However, the application of 1 mM GDP, a non-specific inhibitor of UCP1, reduced this effect by approximately 90%. The significantly reduced proton current (approximately 75%) in UCP1 knockout mitoplasts further confirmed that UCP1 plays a primary role in mediating the proton leak induced by DNP in wild-type mitochondria. Interestingly, the data point to the low potency of DNP in mitoplasts, evidenced by the need for relatively high concentrations (50–500 µM) to induce a significant H⁺ conductance change.

Similar experiments focusing on the effect of DNP on ANT1 were performed using mitoplasts from skeletal muscle (SkM) and heart, where ANT1 is the dominant isoform. ANT1 was considered a likely candidate for potentiating DNP-mediated uncoupling, as previous work by the authors demonstrated that ANT1 enhances fatty acid – induced proton current in the IMM (Bertholet et al. 2019). Patch-clamp recordings revealed a DNP concentration-dependent increase in proton current in the heart IMM. The application of CATR reduced the proton leak by 60%, suggesting that ANT1 plays a predominant role in DNP-mediated uncoupling in heart mitochondria. Measurements of the significant proton current in ANT1 KO heart mitochondria and the lack of sensitivity to CATR suggest possible activation of other proteins from the IMM (for example UCP3 and OGC). Although the amount of each protein may be lower than that of ANT, the presence of several DNP-sensitive proteins could explain residual conductance. Unfortunately, the authors did not explore this issue further. Here, additional experiments with e.g. GDP or ATP would help to see the involvement of UCP3 in the observed proton current (Pohl et al. 2019; Macher et al. 2018).

On the other hand, since the site where CATR binds to ANT1 was previously known (Pebay-Peyroula et al. 2003; Nury et al. 2005; Ruprecht et al. 2014), the efficient inhibition of DNP action by CATR may indicate that DNP interacts with the same amino acid residues in ANT1. The authors use this as key evidence to propose an explanation for the molecular mechanism of DNP’s action on ANT1. Taken together, the residual proton current in the UCP1-KO and ANT1-KO mitoplasts indicates that DNP may activate other proteins in the IMM as well.

The major limitation of this method is that it can only detect protein activation when the protein is overexpressed in the mitochondria. The effect of DNP on all other components of the IMM is therefore hidden and subject to speculation. This means that experiments will be limited on cardiac and BAT mitochondria in future studies.

There is also the problem of the interaction of DNP with other lipophilic substrates. For example, FA scavengers attenuate the effect of DNP. Thus, the concentration at which DNP induces a proton current resulting from UCP1 activation remains unknown. 

### Insights from molecular dynamic simulations

In silico analysis provides valuable insights into the molecular interactions between substrates and membrane components, such as proteins and lipids. ANT is a favorable candidate for this analysis because its crystallographic structure has been available since 2003 (Pebay-Peyroula et al. 2003; Nury et al. 2005; Ruprecht et al. 2014). Structural studies have also identified the internal cavity of ANT1 as the site where transported nucleotides, such as ATP and ADP, interact with the protein (Kunji and Robinson 2006; Dehez et al. 2008).

**Fig. 5:**
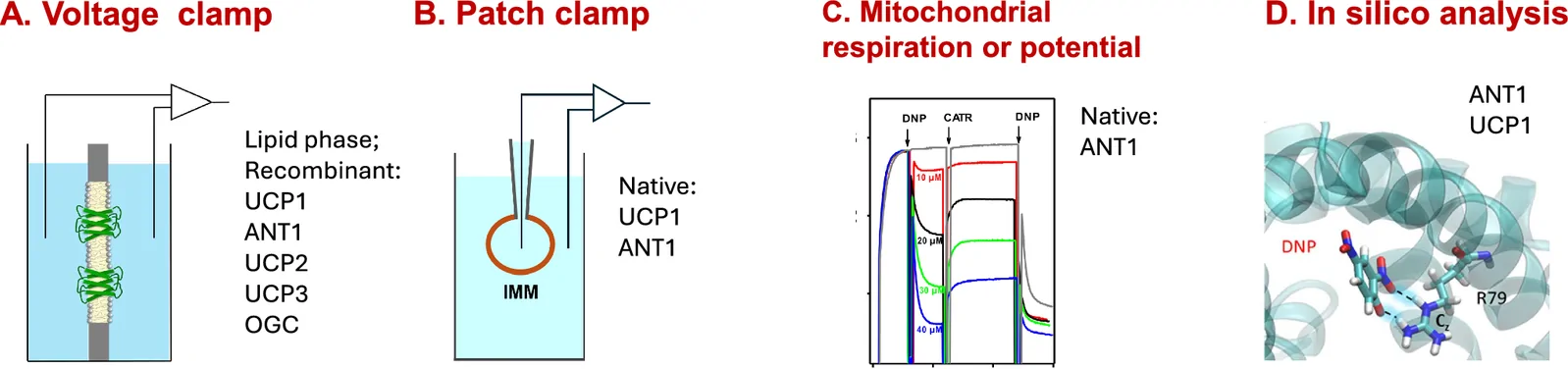
A comparison of methods for studying the interaction between DNP and mitochondrial proteins and lipids. **A**. Conductance measurements using planar bilayer membranes: well-defined buffer and lipid composition, contribution only from target protein. **B**. The patch-clamp technique on mitoplasts enables measurements of native proteins in the IMM. **C**. Respiration/potential experiments: enable measurements in cells. **D**. In silico methods (molecular docking and dynamics simulation): putative electrostatic interactions and visualization are possible. Figures **C** and **D** have been adapted from Zuna et al., 2021

Based on experimental results that demonstrated the importance of the R79 residue for ANT`s ability to mediate DNP-induced proton conductance in planar lipid membranes, Žuna et al. applied MD simulations to explore the interaction between the R79 and the anion form of DNP (Zuna et al. 2021). The MD simulations, conducted over 500 ns focused on two systems: wild-type ANT1 and ANT1-R79S mutant, where arginine was substituted for serine at position 79. The results revealed that the DNP anion readily binds to R79 in ANT1, while binding was absent in the ANT1-R79S. The relatively large average distance (1 nm) between the center of mass of the DNP and the C_Z_ atom in R79 (Fig. 5 from (Zuna et al. 2021)) suggests that the interaction is not particularly strong or long-lasting. This observation supports the hypothesis that the mechanism of DNP action may be similar to the FA sliding hypothesis (Kreiter et al. 2023; Vojvodic et al. 2025) (Fig. 4C). By contrast, particularly strong and long-lasting interactions between the DNP anion and amino acid residues would suggest the permanent presence of the DNP anion in the protein cavity.

To identify possible binding sites for DNP within the cavity of ANT1, Bertholet et al. applied both, in silico docking and MD simulations (Bertholet et al. 2022). Docking of DNP in the c-state, based on the bovine ANT1 structure, revealed three main types of interactions between the DNP anion and cavity residues: (i) hydrophobic interactions, where the aromatic moiety of DNP interacts with the hydrophobic side chains of I183 and Y186, (ii) electrostatic interactions between the nitro group on DNP and R234, and (iii) hydrogen bond interactions between the DNP hydroxyl group and S227. Docking revealed that the DNP anion occupies a position in the cavity similar to that of the adenosine ring of ADP or the diterpene moiety of CATR (Bertholet et al. 2022).

The authors did not delve into the specific amino acid environment that contribute to the stabilization of the DNP anion, although their results were somewhat counterintuitive. One might expect the negatively charged hydroxyl group of DNP to orient toward the positively charged R234, and the slightly polar-NO_2_ group to turn toward the polar serine. However, the authors observed that the DNP anion adopts highly reproducible binding modes in the c-state cavity, which were notably unaffected by the protonation state of DNP. Additionally, docking of DNP in the m-state, based on the fungal ANT1 structure (Ruprecht et al. 2019), revealed that DNP localizes in the same position within the cavity, surrounded by the same residues as in the c-state, which was obtained for the bovine model. Therefore, the authors argued that their in silico findings support the experimental observation that ANT1 facilitates DNP-mediated proton leak in both the c- and m-states. Further docking experiments with other uncouplers, including FCCP, BAM15, and SF6487, in the c-state cavity showed that they all bind to the same pocket identified for DNP, as well as for m-state (Bertholet et al. 2022).

MD simulations confirmed the docking predictions, revealing that within 100 ns, the DNP anion, initially located away from the protein, was drawn toward the predicted binding site by the strong positive potential in the cavity (Bertholet et al. 2022). Further, 500 ns simulations demonstrated that DNP remained bound at this predicted location, even at a transmembrane potential of -160 mV, maintaining its docking interactions and interacting with residues R79, R187, and D231. Additional MD simulations with the only known natural uncoupler, FA (arachidonic or palmitic acid), showed that FAs bind to the water-filled ANT cavity, but to a different site than DNP. In 7 out of 10 simulations in c-state, FAs entered the protein between TM5 and TM6, where they remained, interacting with residues K22, R79, and R279, while the acyl chain extended into the lipid bilayer.

In summary, MD simulations of both natural and chemical uncouplers demonstrated that the carboxylic group of FAs interacts with residues involved in nucleotide phosphate group binding (Kunji and Robinson 2006; Dehez et al. 2008), whereas the DNP binding site overlaps with the CATR binding site and the localization of the adenosine ring of ADP. In both cases, the protonable groups of these uncouplers are located at the bottom of the cytosolic cavity. Based on these in silico results, the authors proposed a three-step mechanism for proton permeation mediated by ANT and uncouplers: (i) FA and DNP bind to the c-state of ANT, reducing the positive potential at the bottom of the cavity, (ii) this allows cytoplasmic H^+^ to enter the cavity and bind to the uncoupler, and (iii) the binding of FA or DNP induces a slight conformational change from the c- to the m-state, opening a narrow, selective pathway for H^+^ permeation (Bertholet et al. 2022).

Finally, a mathematical model was developed to investigate the potential for H^+^ permeation through ANT when a DNP-like uncoupler binds to the cavity, opening a proton-selective pathway toward the matrix side (Bertholet et al. 2022). The model supported the authors´ hypothesis, predicting an H^+^ flux rate of approximately 15 to 60 H^+^ per second at transmembrane potential of -160 mV.

It is important to note that the presumed binding sites for uncouplers must be tested in experimental systems by site-directed mutagenesis. Additionally, in silico mutagenesis combined with further docking studies involving uncouplers could provide valuable, complementary insights. In the absence of such experimental data, however, the hypothesis remains primarily based on findings derived from ANT1 and UCP1.

### Insights from cryo-electron structure of UCP1

The recently solved structures of human UCP1, obtained in the nucleotide-free, DNP-bound, and ATP-bound states (Kang and Chen 2023), has provided new insights into potential substrate-protein interactions. Using cryo-EM density data of DNP (added in concentration 10 mM) into the UCP1 cavity, the authors modeled the binding location of DNP within the protein. They found that DNP binds to the positively charged central cavity of UCP1 in the c-state conformation. Four distinct types of interactions between DNP`s functional groups and UCP1 residues were identified: (i) hydrophobic π – π interactions between phenyl ring of DNP and W280 of TM6, (ii) a hydrogen bond interaction between the hydroxyl group of DNP and R276 of TM6, (iii) a combination of polar and hydrophobic interactions between the first nitro group of DNP and N281 and L277 of TM6, and (iv) polar and electrostatic interactions between second nitro group of DNP and R91, Q84, and R83 of TM2 (Kang and Chen 2023). However, the extensive structural analysis could not provide a clear model for DNP-assisted proton transport in UCP1. First, no significant conformational change was observed when comparing the DNP-bound UCP1 state with the empty UCP1 structure. This suggests that, upon DNP binding to the central cavity of UCP1, the gate to the matrix side remains tightly closed, maintaining the same conformation as observed in the empty UCP1 or ATP-bound states. Although Kang and Chen favored the model proposed by Bertholet et al. for the ANT1 (Bertholet et al. 2019), they could not explain how protons could pass through the closed matrix gate. No chain of water molecules, which might facilitate proton movement, was observed, nor were any protonatable residues identified that could allow unhindered proton movement through the gate. The authors admit that, while thermodynamic fluctuations could temporarily open the matrix gate, the precise proton-conducting pathway remains elusive. Consequently, they concluded that the DNP-bound UCP1 structure they obtained represents a non-conducting protein state (Kang and Chen 2023).

## Conclusion

Although DNP has long been used as a model uncoupler, its precise mechanism of action has only recently been revisited with modern techniques (Fig. 5). Earlier mechanistic studies focused on its protonophore activity in pure lipid bilayers, but growing evidence now points to a more complex interaction involving mitochondrial proteins, such as UCPs, ANT1, and OGC. Voltage- and patch-clamp experiments have shown that these proteins facilitate DNP-mediated proton transport in a tissue-specific manner, confirmed by knockout models and inhibitor studies. Mutational analysis, particularly involving ANT1 R79S, and in silico modeling have further highlighted key residues and binding sites involved in DNP interaction. Cryo-EM Structures of UCP1 with DNP have revealed overlapping binding sites with ATP and GDP, supporting competitive inhibition, though the exact proton pathway remains unresolved. Collectively, these findings suggest a paradigm shift: DNP uncoupling is not purely lipid-mediated, but also protein-facilitated. A mechanistic framework that integrates electrophysiology, structural biology, and computational modeling now offers an opportunity to redefine mitochondrial uncoupling beyond empirical pharmacology. Elucidating how DNP engages specific inner mitochondrial membrane proteins and lipid environments may enable the rational design of safer, tunable uncouplers and, critically, mechanism-based antidotes. Such advances could reopen the therapeutic exploration of controlled mitochondrial uncoupling for metabolic diseases while establishing clearer principles for balancing efficacy and safety.

## Abbreviations

ATP: Adenosine-5’-triphosphate
BAT: Brown adipose tissue
BLM: Bilayer lipid membrane
CATR: Carboxyatractyloside
CD: Cyclodextrin
DNP: 2,4-Dinitrophenol
ETC: Electron transport chain
GDP: Guanosine 5’-diphosphate
IMM: Inner mitochondrial membrane
mTOR: Mammalian target of rapamycin signaling pathway
OxPhos: Oxidative phosphorylation
ROS: Reactive oxygen species
TM: Transmembrane helix

## References

[CR1] Abdelati A, Burns MM, Chary M (2023) Sublethal toxicities of 2,4-dinitrophenol as inferred from online self-reports. PLoS ONE 18:e0290630. 10.1371/journal.pone.0290630 PMC10499234 37703241

[CR2] Andreyev A et al (1989) The ATP/ADP-antiporter is involved in the uncoupling effect of fatty acids on mitochondria. Eur J Biochem 182:585–592. 10.1111/j.1432-1033.1989.tb14867.x 2546761

[CR3] Bartlett J, Brunner M, Gough K (2010) Deliberate poisoning with dinitrophenol (DNP): an unlicensed weight loss pill. Emerg Med J 27:159–160. 10.1136/emj.2008.069401 20156878

[CR4] Beck V et al (2006) A new automated technique for the reconstitution of hydrophobic proteins into planar bilayer membranes. Studies of human recombinant uncoupling protein 1. Biochim Biophys Acta 1757:474–479. 10.1016/j.bbabio.2006.03.006 16626624

[CR5] Beck V et al (2007) Polyunsaturated fatty acids activate human uncoupling proteins 1 and 2 in planar lipid bilayers. FASEB J 21:1137–1144. 10.1096/fj.06-7489com 17242157

[CR6] Bertholet AM et al (2019) H(+) transport is an integral function of the mitochondrial ADP/ATP carrier. Nature 571:515–520. 10.1038/s41586-019-1400-3 PMC6662629 31341297

[CR7] Bertholet AM et al (2022) Mitochondrial uncouplers induce proton leak by activating AAC and UCP1. Nature 606:180–187. 10.1038/s41586-022-04747-5 PMC9646675 35614225

[CR8] Bielawski J, Thompson TE, Lehninger AL (1966) The effect of 2,4-dinitrophenol on the electrical resistance of phospholipid bilayer membranes. Biochem Biophys Res Commun 24:948–954. 10.1016/0006-291x(66)90342-1 5970528

[CR9] Cefalu WT et al (2015) Effects of canagliflozin on body weight and relationship to HbA1c and blood pressure changes in patients with type 2 diabetes. Diabetologia 58:1183–1187. 10.1007/s00125-015-3547-2 PMC4800739 25813214

[CR10] Chan SL et al (2006) Mitochondrial uncoupling protein-4 regulates calcium homeostasis and sensitivity to store depletion-induced apoptosis in neural cells. J Biol Chem 281:37391–37403. 10.1074/jbc.M605552200 17035241

[CR11] Cutting WC, Mehrtens HG, Tainter ML (1933) Actions and uses of dinitrophenol: promising metabolic applications. JAMA 101(3):193–195

[CR12] Cypess AM et al (2025) Emerging debates and resolutions in brown adipose tissue research. Cell Metab 37:12–33. 10.1016/j.cmet.2024.11.002 PMC11710994 39644896

[CR13] Dehez F, Pebay-Peyroula E, Chipot C (2008) Binding of ADP in the mitochondrial ADP/ATP carrier is driven by an electrostatic funnel. J Am Chem Soc 130:12725–12733. 10.1021/ja8033087 18729359

[CR14] Ebert A, Hannesschlaeger C, Goss KU, Pohl P (2018) Passive permeability of planar lipid bilayers to organic anions. Biophys J 115:1931–1941. 10.1016/j.bpj.2018.09.025 PMC6303230 30360927

[CR15] Enebo LB et al (2021) Safety, tolerability, pharmacokinetics, and pharmacodynamics of concomitant administration of multiple doses of cagrilintide with semaglutide 2.4 mg for weight management: a randomised, controlled, phase 1b trial. Lancet 397:1736–1748. 10.1016/S0140-6736(21)00845-X 33894838

[CR16] Fedorenko A, Lishko PV, Kirichok Y (2012) Mechanism of fatty-acid-dependent UCP1 uncoupling in brown fat mitochondria. Cell 151:400–413. 10.1016/j.cell.2012.09.010 PMC3782081 23063128

[CR17] Finkelstein A (1970) Weak-acid uncouplers of oxidative phosphorylation. Mechanism of action on thin lipid membranes. Biochim Biophys Acta 205:1–6. 10.1016/0005-2728(70)90055-1 5439517

[CR18] Ginsburg H, Stark G (1976) Facilitated transport of di- and trinitrophenolate ions across lipid membranes by valinomycin and nonactin. Biochim Biophys Acta 455:685–700. 10.1016/0005-2736(76)90041-9 1036715

[CR19] Goldgof M et al (2014) The chemical uncoupler 2,4-dinitrophenol (DNP) protects against diet-induced obesity and improves energy homeostasis in mice at thermoneutrality. J Biol Chem 289:19341–19350. 10.1074/jbc.M114.568204 PMC4094046 24872412

[CR20] Grundlingh J, Dargan PI, El-Zanfaly M, Wood DM (2011) 2,4-dinitrophenol (DNP): a weight loss agent with significant acute toxicity and risk of death. J Med Toxicol 7:205–212. 10.1007/s13181-011-0162-6 PMC3550200 21739343

[CR21] Gummesson A, Zack F, Buettner A (2025) 2,4-dinitrophenol intoxication and its morphological findings as an indication of substance intake. J Pharm Biomed Anal 252:116498. 10.1016/j.jpba.2024.116498 39378760

[CR22] Gziut T, Thomas SHL (2022) International trends in systemic human exposures to 2,4 dinitrophenol reported to poisons centres. Clin Toxicol (Phila) 60:628–631. 10.1080/15563650.2021.2005797 34812657

[CR23] Hanstein WG, Hatefi Y (1974) Characterization and localization of mitochondrial uncoupler binding sites with an uncoupler capable of photoaffinity labeling. J Biol Chem 249:1356–1362 4817750

[CR24] Heidkamper D, Muller V, Nelson DR, Klingenberg M (1996) Probing the role of positive residues in the ADP/ATP carrier from yeast. The effect of six arginine mutations on transport and the four ATP versus ADP exchange modes. Biochemistry 35:16144–16152. 10.1021/bi960668j 8973186

[CR25] Hermetet C et al (2024) Case report: fatal long-term intoxication by 2,4-dinitrophenol and anabolic steroids in a young bodybuilder with muscle dysmorphia. Front Public Health 12:1452196. 10.3389/fpubh.2024.1452196 PMC11628266 39659715

[CR26] Holborow A, Purnell RM, Wong JF (2016) Beware the yellow slimming pill: fatal 2,4-dinitrophenol overdose. BMJ Case Rep. 10.1136/bcr-2016-214689 PMC4840695 27045052

[CR27] Hopfer U, Lehninger AL, Thompson TE (1968) Protonic conductance across phospholipid bilayer membranes induced by uncoupling agents for oxidative phosphorylation. Proc Natl Acad Sci U S A 59:484–490. 10.1073/pnas.59.2.484 PMC224698 5238978

[CR28] Hopfer U, Lehninger AL, Lennarz WJ (1970) The effect of the polar moiety of lipids on bilayer conductance induced by uncouplers of oxidative phosphorylation. J Membr Biol 3:142–155. 10.1007/BF01868012 24174190

[CR29] Hubbard WB et al (2023) Mitochondrial dysfunction after repeated mild blast traumatic brain injury is attenuated by a mild mitochondrial uncoupling prodrug. J Neurotrauma 40:2396–2409. 10.1089/neu.2023.0102 PMC10653072 37476976

[CR30] Jovanovic O, Gille L, Vazdar M, Pohl EE (2019) Membrane lipids alter uncoupling effect of 2,4 dinitrophenol. Biophys J 116:511a–511a. 10.1016/j.bpj.2018.11.2754

[CR31] Kang Y, Chen L (2023) Structural basis for the binding of DNP and purine nucleotides onto UCP1. Nature 620:226–231. 10.1038/s41586-023-06332-w 37336486

[CR32] Kopec KT, Kim T, Mowry J, Aks S, Kao L (2019) Role of dantrolene in dinitrophenol (DNP) overdose: a continuing question? Am J Emerg Med 37:1216 e1211-1216 e1212. 10.1016/j.ajem.2019.03.035 30948257

[CR33] Kotova EA, Antonenko YN (2022) Fifty years of research on protonophores: mitochondrial uncoupling as a basis for therapeutic action. Acta Nat 14:4–13. 10.32607/actanaturae.11610 PMC9013436 35441048

[CR34] Krauss S, Zhang CY, Lowell BB (2005) The mitochondrial uncoupling-protein homologues. Nat Rev Mol Cell Biol 6:248–261. 10.1038/nrm1592 15738989

[CR35] Kreiter J et al (2021) ANT1 activation and inhibition patterns support the fatty acid cycling mechanism for proton transport. Int J Mol Sci. 10.3390/ijms22052490 PMC7958136 33801254

[CR36] Kreiter J et al (2023) FA sliding as the mechanism for the ANT1-mediated fatty acid anion transport in lipid bilayers. Int J Mol Sci 24:13701. 10.3390/ijms241813701 PMC10531397 37762012

[CR37] Kunji ER, Robinson AJ (2006) The conserved substrate binding site of mitochondrial carriers. Biochim Biophys Acta 1757:1237–1248. 10.1016/j.bbabio.2006.03.021 16759636

[CR38] Kushner RF et al (2020) Semaglutide 2.4 mg for the treatment of obesity: key elements of the STEP trials 1 to 5. Obesity 28:1050–1061. 10.1002/oby.22794 PMC7318657 32441473

[CR39] Lea EJ, Croghan PC (1969) The effect of 2,4-dinitrophenol on the properties of thin phospholipid films. J Membr Biol 1:225–237. 10.1007/BF01869783 24174051

[CR40] Leblanc OH Jr. (1971) The effect of uncouplers of oxidative phosphorylation on lipid bilayer membranes: Carbonylcyanidem-chlorophenylhydrazone. J Membr Biol 4:227–251. 10.1007/BF02431973 24174241

[CR41] Liberman EA, Topaly VP (1968) Selective transport of ions through bimolecular phospholipid membranes. Biochim Biophys Acta 163:125–136. 10.1016/0005-2736(68)90089-8 5686268

[CR42] Lindeman E et al (2026) Runaway uncoupling in 2,4-dinitrophenol poisoning: clinical and mitochondrial observations from two cases. Toxicol Rep 16:102183. 10.1016/j.toxrep.2025.102183 PMC12756549 41487961

[CR43] Liu D et al (2006) Mitochondrial UCP4 mediates an adaptive shift in energy metabolism and increases the resistance of neurons to metabolic and oxidative stress. Neuromolecular Med 8:389–414 10.1385/NMM:8:3:389 16775390

[CR44] Liu D et al (2015) The mitochondrial uncoupler DNP triggers brain cell mTOR signaling network reprogramming and CREB pathway up‐regulation. J Neurochem 134:677–692. 10.1111/jnc.13176 PMC4516713 26010875

[CR45] Loomis WF, Lipmann F (1948) Reversible inhibition of the coupling between phosphorylation and oxidation. J Biol Chem 173:807 18910739

[CR46] Lupsa BC, Inzucchi SE (2018) Use of SGLT2 inhibitors in type 2 diabetes: weighing the risks and benefits. Diabetologia 61:2118–2125. 10.1007/s00125-018-4663-6 30132031

[CR47] Macher G et al (2018) Inhibition of mitochondrial UCP1 and UCP3 by purine nucleotides and phosphate. Biochimica Et Biophysica Acta (BBA) - Biomembranes 1860:664–672. 10.1016/j.bbamem.2017.12.001 PMC6118327 29212043

[CR48] Madeiro da Costa RF, Blanco Martinez AM, Ferreira ST (2010) 2, 4-Dinitrophenol blocks neurodegeneration and preserves sciatic nerve function after trauma. J Neurotrauma 27:829–841 10.1089/neu.2009.1189 20143955

[CR49] McLaughlin S (1972) The mechanism of action of DNP on phospholipid bilayer membranes. J Membr Biol 9:361–372. 10.1007/BF01868062 4640973

[CR50] McLaughlin SG, Dilger JP (1980) Transport of protons across membranes by weak acids. Physiol Rev 60:825–863. 10.1152/physrev.1980.60.3.825 6248908

[CR51] Mitchell P (1966) Chemiosmotic coupling in oxidative and photosynthetic phosphorylation. Biol Rev Camb Philos Soc 41:445–502. 10.1111/j.1469-185x.1966.tb01501.x 5329743

[CR52] Mitchell P, Moyle J (1967a) Respiration-driven proton translocation in rat liver mitochondria. Biochem J 105:1147–1162. 10.1042/bj1051147 PMC1198436 16742541

[CR53] Mitchell P, Moyle J (1967b) Acid-base titration across the membrane system of rat-liver mitochondria. Catalysis by uncouplers. Biochem J 104:588–600. 10.1042/bj1040588 PMC1270625 6048801

[CR54] Myerson M, Paparodis RD (2024) Pharmacotherapy of weight-loss and obesity with a focus on GLP 1-receptor agonists. J Clin Pharmacol 64:1204–1221. 10.1002/jcph.2487 38924121

[CR55] Nelson DR, Lawson JE, Klingenberg M, Douglas MG (1993) Site-directed mutagenesis of the yeast mitochondrial ADP/ATP translocator. Six arginines and one lysine are essential. J Mol Biol 230:1159–1170. 10.1006/jmbi.1993.1233 8487299

[CR56] Nicholls DG (2021) Mitochondrial proton leaks and uncoupling proteins. Biochim Biophys Acta Bioenerg 1862:148428. 10.1016/j.bbabio.2021.148428 33798544

[CR57] Nicholls DG (2023) Fifty years on: how we uncovered the unique bioenergetics of brown adipose tissue. Acta Physiol (Oxf) 237:e13938. 10.1111/apha.13938 36692160

[CR58] Nury H et al (2005) Structural basis for lipid-mediated interactions between mitochondrial ADP/ATP carrier monomers. FEBS Lett 579:6031–6036. 10.1016/j.febslet.2005.09.061 16226253

[CR59] O’Neil PM et al (2018) Efficacy and safety of semaglutide compared with liraglutide and placebo for weight loss in patients with obesity: a randomised, double-blind, placebo and active controlled, dose-ranging, phase 2 trial. Lancet 392:637–649. 10.1016/S0140-6736(18)31773-2 30122305

[CR60] O’Neill N et al (2025) Mitochondrial uncoupler, 2,4-dinitrophenol, reduces spinal cord paralysis and retinal ganglion cell loss in the experimental autoimmune encephalomyelitis model of multiple sclerosis. Biomolecules. 10.3390/biom15020189 PMC11852757 40001492

[CR61] Ost M, Keipert S, Klaus S (2017) Targeted mitochondrial uncoupling beyond UCP1 - the fine line between death and metabolic health. Biochimie 134:77–85. 10.1016/j.biochi.2016.11.013 27916644

[CR62] Pandya JD et al (2007) Post-injury administration of mitochondrial uncouplers increases tissue sparing and improves behavioral outcome following traumatic brain injury in rodents. J Neurotrauma 24:798–811 10.1089/neu.2006.3673 17518535

[CR63] Parker VH (1965) Uncouplers of rat-liver mitochondrial oxidative phosphorylation. Biochem J 97:658–662. 10.1042/bj0970658 PMC1264742 5881655

[CR64] Pebay-Peyroula E et al (2003) Structure of mitochondrial ADP/ATP carrier in complex with carboxyatractyloside. Nature 426:39–44. 10.1038/nature02056 14603310

[CR65] Perry RJ, Zhang D, Zhang XM, Boyer JL, Shulman GI (2015) Controlled-release mitochondrial protonophore reverses diabetes and steatohepatitis in rats. Science 347:1253–1256. 10.1126/science.aaa0672 PMC4495920 25721504

[CR66] Pohl EE, Rupprecht A, Macher G, Hilse KE (2019) Important trends in UCP3 investigation. Front Physiol 10:470. 10.3389/fphys.2019.00470 PMC6524716 31133866

[CR67] Pohl EE, Vazdar M, Kreiter J (2025) Exploring the proton transport mechanism of the mitochondrial ADP/ATP carrier: FA-cycling hypothesis and beyond. Protein Sci 34:e70047. 10.1002/pro.70047 PMC11837047 39969060

[CR68] Rognstad R, Katz J (1969) The effect of 2,4-dinitrophenol on adipose-tissue metabolism. Biochem J 111:431–444. 10.1042/bj1110431 PMC1187562 4388239

[CR69] Roticiani G, Kreiter J, Pohl EE (2025) The non-thermogenic function of opossum UCP1 is independent of its cytoplasmic binding network. bioRxiv. 10.1101/2025.04.27.650829

[CR70] Rupprecht A et al (2012) Quantification of uncoupling protein 2 reveals its main expression in immune cells and selective up-regulation during T-cell proliferation. PLoS ONE 7:e41406. 10.1371/journal.pone.0041406 PMC3411681 22870219

[CR71] Rupprecht A et al (2014) Uncoupling protein 2 and 4 expression pattern during stem cell differentiation provides new insight into their putative function. PLoS ONE 9:e88474. 10.1371/journal.pone.0088474 PMC3921169 24523901

[CR72] Ruprecht JJ et al (2014) Structures of yeast mitochondrial ADP/ATP carriers support a domain-based alternating-access transport mechanism. Proc Natl Acad Sci USA 111:E426-434. 10.1073/pnas.1320692111 PMC3910652 24474793

[CR73] Ruprecht JJ et al (2019) The molecular mechanism of transport by the mitochondrial ADP/ATP carrier. Cell 176:435-447 e415. 10.1016/j.cell.2018.11.025 PMC6349463 30611538

[CR74] Sanjari M, Hadavizadeh M, Sadeghi N, Naghibzadeh-Tahami A (2025) Effect of empagliflozin on weight in patients with prediabetes and diabetes. Sci Rep 15:118. 10.1038/s41598-024-83820-7 PMC11696924 39748045

[CR75] Scott LJ (2015) Liraglutide: a review of its use in the management of obesity. Drugs 75:899–910. 10.1007/s40265-015-0408-8 25985864

[CR76] Shabalina IG et al (2013) UCP1 in brite/beige adipose tissue mitochondria is functionally thermogenic. Cell Rep 5:1196–1203. 10.1016/j.celrep.2013.10.044 24290753

[CR77] Shabalina IG, Jimenez B, Sousa-Filho CPB, Cannon B, Nedergaard J (2025) In isolated brown adipose tissue mitochondria, UCP1 is not essential for - nor involved in - the uncoupling effects of the classical uncouplers FCCP and DNP. Biochim Biophys Acta Bioenerg 1866:149516. 10.1016/j.bbabio.2024.149516 39357779

[CR78] Siegmueller C, Narasimhaiah R (2010) ‘Fatal 2,4-dinitrophenol poisoning coming to a hospital near you.’ Emerg Med J 27:639–640. 10.1136/emj.2009.072892 20511642

[CR79] Silver HJ et al (2023) Effect of the glucagon-like peptide-1 receptor agonist liraglutide, compared to caloric restriction, on appetite, dietary intake, body fat distribution and cardiometabolic biomarkers: a randomized trial in adults with obesity and prediabetes. Diabetes Obes Metab 25:2340–2350. 10.1111/dom.15113 PMC10544709 37188932

[CR80] Skulachev VP (1998) Uncoupling: new approaches to an old problem of bioenergetics. Biochim Biophys Acta 1363:100–124. 10.1016/s0005-2728(97)00091-1 9507078

[CR81] Skulachev VP et al (1968) The effect of uncouplers on mitochondria, respiratory enzyme complexes and artificial phospholipid membranes. Curr Mod Biol 2:98–105. 10.1016/0303-2647(68)90014-2 5650002

[CR82] Vanthourenhout S et al (2026) Further elucidation of the metabolism of 2,4-dinitrophenol in a case of unexpected death. J Anal Toxicol. 10.1093/jat/bkag003 41530400

[CR83] Vlasova KY et al (2022) Liposomal form of 2,4-dinitrophenol lipophilic derivatives as a promising therapeutic agent for ATP synthesis inhibition. Nanomaterials (Basel). 10.3390/nano12132162 PMC9268429 35808003

[CR84] Vojvodic S, Roticiani G, Vazdar M, Pohl EE (2025) Molecular dynamics simulations of a putative novel mechanism for UCP1-assisted FA anion transport. Acta Physiol (Oxf) 241:e70068. 10.1111/apha.70068 PMC12153029 40497434

[CR85] Watanabe JH, Kwon J, Nan B, Reikes A (2024) Trends in glucagon-like peptide 1 receptor agonist use, 2014 to 2022. J Am Pharm Assoc (2003) 64:133–138. 10.1016/j.japh.2023.10.002 37821008

[CR86] Wise J (2014) Increase in reports of toxicity from fat burning supplement seen in UK. Bmj-British Medical Journal. 10.1136/bmj.g4188 24963061

[CR87] Yen M, Ewald MB (2012) Toxicity of weight loss agents. J Med Toxicol 8:145–152. 10.1007/s13181-012-0213-7 PMC3550246 22351299

[CR88] Zackova M, Skobisova E, Urbankova E, Jezek P (2003) Activating omega-6 polyunsaturated fatty acids and inhibitory purine nucleotides are high affinity ligands for novel mitochondrial uncoupling proteins UCP2 and UCP3. J Biol Chem 278:20761–20769. 10.1074/jbc.M212850200 12670931

[CR89] Zhong R et al (2025) Micro‐doses of DNP preserve motor and muscle function with a period of functional recovery in amyotrophic lateral sclerosis mice. Ann Neurol 97:542–557. 10.1002/ana.27140 PMC11831883 39552508

[CR90] Zuna K et al (2021) Mitochondrial uncoupling proteins (UCP1-UCP3) and adenine nucleotide translocase (ANT1) enhance the protonophoric action of 2,4-dinitrophenol in mitochondria and planar bilayer membranes. Biomolecules. 10.3390/biom11081178 PMC8392417 34439844

[CR91] Zuna K et al (2024) The 2-oxoglutarate/malate carrier extends the family of mitochondrial carriers capable of fatty acid and 2,4-dinitrophenol-activated proton transport. Acta Physiol (Oxf) 240:e14143. 10.1111/apha.14143 PMC11475482 38577966

